# Supplementary material for: Single-cell analysis of transcription kinetics across the cell cycle
Source: eLife. 2016 Jan 29;5:e12175. doi: 10.7554/eLife.12175 (PMC4801054; doi:10.7554/eLife.12175)
Supplement: Supplementary file 1. — DOI: http://dx.doi.org/10.7554/eLife.12175.017 [file elife-12175-supp1.docx]

**Supplementary file 1. Literature estimates of transcription parameters used in study.**

| **A. Literature estimates of numbers of mRNA and active transcription sites (TS) per cell for *Oct4* and *Nanog*.** | | | | | | | |
| --- | --- | --- | --- | --- | --- | --- | --- |
| **Reference** | ***Oct4,***  **#mRNA/cell,**  **mean** | ***Oct4,***  **#mRNA/cell,**  **CV^†^** | ***Oct4,***  **#TS/cell,**  **mean** | ***Nanog,***  **#mRNA/cell,**  **mean** | ***Nanog,***  **#mRNA/cell,**  **CV^†^** | ***Nanog,***  **#TS/cell,**  **mean** | **Cell line** |
| Ochiai et al.  (2014) |  |  |  | 133 | 0.85 |  | WT (C57BL/6) |
|  |  |  |  | 77 | 0.89 | 0.02* | Reporter |
| Abranches et al.  (2014) |  |  |  | 94 | 1.07 |  | WT (E14Tg2A) |
|  |  |  |  | 111 | 0.96 |  | Reporter |
| Singer et al.  (2014) | 125* | 0.45* |  | 100* | 0.85* |  | WT (E14Tg2A) |
| Grün et al.  (2014) | 150* | 0.3* |  |  |  |  | WT (J1) |
| Faddah et al.  (2013) | 250* |  |  | 125* |  |  | WT (E14Tg2a) |
|  | 195* |  |  | 105* |  |  | WT (V6.5) |
|  | 175* |  |  | 105* |  |  | Hybrid (ESC1) |
|  | 270* |  |  | 210* |  |  | Hybrid (F1-2-1) |
|  | 180* |  |  | 60* |  |  | WT (4.7-Balb/c) |
|  | 195* |  |  | 110* |  |  | WT (C57BL/6) |
|  | 160* | 0.4* |  | 110* | 0.6* |  | Reporter |
| Muñoz Descalzo et al.  (2013) |  |  |  | 200* |  |  | WT (E14Tg2A) |
| Hansen et al.  (2013) |  |  |  | 221 |  | 1.1* | Hybrid (2-1) |
| **Literature mean ± SEM** | **189**  **±15** | **0.38**  **±0.08** |  | **126**  **±13** | **0.89**  **±0.07** | **0.61**  **±0.45** |  |
| **This study mean ± SEM** | **477**  **±67** | **0.34**  **±0.01** | **0.79**  **±0.12** | **125**  **±24** | **0.80**  **±0.05** | **0.60**  **±0.01** | WT (R1) |

All experiments were performed under serum/LIF growth conditions.
**^†^** - Coefficient of variation (CV)
* - Value estimated from figure

| **B. Literature estimates of burst frequency, burst size, and transcription initiation rate for *Oct4* and *Nanog*.** | | | | | | | | |
| --- | --- | --- | --- | --- | --- | --- | --- | --- |
| **Reference** | ***Oct4* burst frequency^1^**  **(min^-1^)** | ***Oct4* burst size^2^**  **(#mRNA/burst)** | ***Oct4* transcription initiation rate^3^ (min^-1^)** | ***Nanog* burst frequency^1^**  **(min^-1^)** | ***Nanog* burst size^2^**  **(#mRNA/burst)** | ***Nanog* transcription initiation rate^3^**  **(min^-1^)** | **Cell line** |  |
| Ochiai et al.  (2014) |  |  |  | 2.82 × 10^-2^ | 3.5 | 2.11 | Reporter |  |
| Singer et al.  (2014) | 8.7 × 10^-3^ | 87 |  | 6.5 × 10^-3^ | 33 |  | WT (E14Tg2A) |  |
| **This study** | **9.2 × 10^-3^** | **106** | **1.9** | **1.9 × 10^-3^** | **123** | **0.85** | **WT (R1)** |  |

Our estimated parameters are consistent with previously reported values. Specifically: (i) The rates of gene activation for both genes (*Oct4*: *k*_ON_=9.2×10^-3^±1.4×10^-3^ min^-1^; *Nanog*: *k*_ON_=1.9×10^-3^±0.2×10^-3^ min^-1^; 3 experiments for each gene, with >600 cells per experiment) are comparable to the ones recently estimated by (Singer et al., 2014). These authors also described a slower switching process between *Nanog* activity states, which we did not observe. This could be due to either a difference in experimental conditions or due to the lower number of cells in our sample.

All experiments were performed under serum/LIF growth conditions.
^1^ - Burst frequency = *k*_ON_.
^2^ - Burst size = *k*_INI_/*k*_OFF_.
^3^ - Transcription initiation rate = *k*_INI_

| **C. Literature estimates of transcription initiation rate, elongation rate, and splicing delay time in mammalian genes.** | | | | | |
| --- | --- | --- | --- | --- | --- |
| **Reference** | **Transcription initiation rate (min^-1^)** | **Elongation rate**  **(kb/min)** | **Splicing delay**  **(min)** | **Sample** |  |
| Bahar Halpern et al.  (2015) | 0.06-1.7 |  |  | Mouse liver tissue |  |
| Coulon et al.  (2014) |  | 1.44-2.64 | 4.2-11.6 | Human osteosarcoma cells  (U20S) |  |
| Jonkers et al.  (2014) |  | 0.5-4 |  | Mouse embryonic stem cells  (V6.5) |  |
| Hoyle and Ish-Horowicz  (2013) |  | 3.1-4.0 | 9.7-12.5 | Mouse presomitic mesoderm tissue |  |
| Martin et al.  (2013) |  | 3-6 | 0.33-0.5 | Human embryonic kidney cells (HEK 293) |  |
| Yunger et al.  (2010) | 1.15-2.73 | 0.31-0.78 |  | Human embryonic kidney cells (HEK 293) |  |
| Singh and Padgett  (2009) |  | 3.8 | 5-10 | Human Tet-21 cells |  |
| Darzacq et al.  (2007) | 1.3 | 4.3 |  | Human osteosarcoma cells  (U20S) |  |
| Audibert et al.  (2002) |  |  | 0.4-7.5 | Mouse embryonic fibroblasts  (NIH-3T3) |  |

The rate of transcription initiation for *Nanog* when in the active state (*k*_INI_=0.85±0.12 min^-1^) is close to the value recently measured using a live mRNA reporter (Ochiai et al., 2014). More broadly, the rates of initiation for both *Oct4* (*k*_INI_=1.9±0.3 min^-1^) and *Nanog* fall within the typical values for mammalian genes, as reported in the literature (0.06-2.73 min^-1^ (Bahar Halpern et al., 2015; Ochiai et al., 2014; Yunger et al., 2010)). The estimated residence times of nascent mRNA (*Oct4*: τ_RES_=3.5±0.7 min, *Nanog*: τ_RES_=7.7±1.7 min) are consistent with the reported values of elongation speed (0.31-6 kb/min (Coulon et al., 2014; Darzacq et al., 2007; Hoyle and Ish-Horowicz, 2013; Jonkers et al., 2014; Martin et al., 2013; Singh and Padgett, 2009; Yunger et al., 2010)) and splicing delay times (0.33-12.5 min (Audibert et al., 2002; Coulon et al., 2014; Hoyle and Ish-Horowicz, 2013; Martin et al., 2013; Singh and Padgett, 2009)). In particular, the longer residence time for *Nanog* is consistent with it being the longer gene of the two (*Oct4*: 4.7 kb, *Nanog*: 7.1 kb).

| **D. mRNA half-life (τ_1/2_) estimates from Sharova et al., 2009** | | | |
| --- | --- | --- | --- |
| **Sample** | ***Oct4* τ_1/2_ (hr)** | ***Nanog* τ_1/2_ (hr)** |  |
| MC1/LIF+ | 4.58 | 5.62 |  |
| MC2/LIF+ | 3.95 | 6.35 |  |
| **Study mean**  **(used in Table S3.2)** | **4.26** | **5.98** |  |

All experiments were performed under serum/LIF growth conditions.

| **E. Literature estimates of mRNA half-life (τ_1/2_)** | | |
| --- | --- | --- |
| **Reference** | ***Oct4* τ_1/2_ (hr)** | ***Nanog* τ_1/2_ (hr)** |
| Ochiai et al.  (2014) |  | 4.72 |
| Abranches et al.  (2013) | 6 | 4.7 |
| Muñoz Descalzo et al.  (2013) | 4.6 | 5.6 |
| Sharova et al.  (2009) | 4.26 | 5.98 |
| **Literature mean**  **(used in model)** | **4.95** | **5.25** |

All experiments were performed under serum/LIF growth conditions.

| **F. Converting literature mean of mRNA half-life (τ_1/2_)**  **to mRNA lifetime (τ) and degradation rate (*k*_D_)** | | | | |
| --- | --- | --- | --- | --- |
| **Parameter** | **Expression** | ***Oct4*** | ***Nanog*** |  |
| Lifetime (τ) | τ = τ_1/2_/log(2) | 7.14 hr | 7.57 hr |  |
| Degradation rate (*k*_D_) | *k*_D_ = log(2)/τ_1/2_ | 0.14 hr^-1^ | 0.13 hr^-1^ |  |
