# Supplementary material for: Single-cell analysis of transcription kinetics across the cell cycle
Source: eLife. 2016 Jan 29;5:e12175. doi: 10.7554/eLife.12175 (PMC4801054; doi:10.7554/eLife.12175)
Supplement: Supplementary file 2. — DOI: http://dx.doi.org/10.7554/eLife.12175.018 [file elife-12175-supp2.docx]

**Supplementary file 2. Estimated parameters of transcription for *Oct4* and *Nanog*.**

| **A. Parameters of transcription for *Oct4* and *Nanog* estimated from the stochastic model.** | | |
| --- | --- | --- |
| **Parameter** | ***Oct4*** | ***Nanog*** |
| Gene activation rate  (*k*_ON_) | 9.2×10^-3^±1.4×10^-3^ min^-1^ | 1.9×10^-3^±0.1×10^-3^ min^-1^ |
| Gene inactivation rate  (*k*_OFF_) | 1.8×10^-2^±0.6×10^-2^ min^-1^ | 6.9×10^-3^±0.5×10^-3^ min^-1^ |
| Transcription initiation rate  (*k*_INI_) | 1.9±0.3 min^-1^ | 0.8±0.1 min^-1^ |
| Nascent mRNA residence time  (τ_RES_) | 3.5±0.7 min | 7.7±1.7 min |
| Fold change in nascent mRNA per gene copy  (*η*) | 0.72±0.05 | 0.76±0.06 |
| Fold change in gene activation rate per gene copy  (*α*) | 0.63±0.06 | 0.71±0.06 |

| **B. Quantities estimated from transcription parameters for *Oct4* and *Nanog* before and after gene replication.** | | | | | | | | | |
| --- | --- | --- | --- | --- | --- | --- | --- | --- | --- |
| **Quantity**  **per gene copy** |  | **Expression used** | |  | ***Oct4*** | |  | ***Nanog*** | |
|  |  | **before** | **after** |  | **before** | **after** |  | **before** | **after** |
| Mean “ON” duration |  | $\frac{1}{k_{OFF}}$ | $\frac{1}{k_{OFF}}$ |  | 56±19 min | 56±19 min |  | 144±11 min | 144±11 min |
| Mean “OFF” duration |  | $\frac{1}{k_{ON}}$ | $\frac{1}{\alpha k_{ON}}$ |  | 108±17 min | 173±31 min |  | 534±28 min | 750±75 min |
| Fraction of time “ON” |  | $\frac{k_{ON}}{k_{ON}+k_{OFF}}$ | $\frac{\alpha k_{ON}}{\alpha k_{ON}+k_{OFF}}$ |  | 0.34±0.08 | 0.24±0.05 |  | 0.22±0.02 | 0.16±0.01 |
| Mean number of mRNA produced during an “ON” period |  | $\frac{k_{INI}}{k_{OFF}}$ | $\frac{k_{INI}}{k_{OFF}}$ |  | 106±39 | 106±39 |  | 123±17 | 123±17 |
