# Supplementary material for: Single-cell analysis of transcription kinetics across the cell cycle
Source: eLife. 2016 Jan 29;5:e12175. doi: 10.7554/eLife.12175 (PMC4801054; doi:10.7554/eLife.12175)
Supplement: Supplementary file 3. — DOI: http://dx.doi.org/10.7554/eLife.12175.019 [file elife-12175-supp3.docx]

| **Supplementary file 3. Sequences of smFISH probes** | |
| --- | --- |
| **Transcript** | **Probe sequences (5’ to 3’)** |
| *Oct4* exons (48 probes) | \| tgtccagccatggggaaggt \| \| --- \| \| tggaggcccttggaagctta \| \| tccctccgcagaactcgtat \| \| tcaggctgcaaagtctccac \| \| ttctccaacttcacggcatt \| \| tcttctgcttcagcagcttg \| \| tggtctggctgaacaccttt \| \| ttcctccacccacttctcca \| \| tagttcgctttctcttccgg \| \| atctgctgtagggagggctt \| \| cgccggttacagaaccatac \| \| ggtgtccctgtagcctcata \| \| gtgtggtgaagtgggggctt \| \| agtttgaatgcatgggagag \| \| ccctcctcagtaaaagaatt \| \| ctcctgatcaacagcatcac \| \| aggttcgaggatccacccag \| \| acatggggagatccccaata \| \| aacttgggggactaggccca \| \| tcagaggaggttccctctga \| \| aactgttctagctccttctg \| \| aaagagaacgcccagggtga \| \| catgttcttaaggctgagct \| \| gtctccgatttgcatatctc \| \| tcagaaacatggtctccaga \| \| gaaccacatccttctctagc \| \| cttctcgttgggaatactca \| \| atagcctggggtgccaaagt \| \| tgacgggaacagagggaaag \| \| aaagctccaggttctcttgt \| \| agcttctttccccatcccac \| \| gtgtgtcccagtctttattt \| \| tgagaaggcgaagtctgaag \| \| tgagcctggtccgattccag \| \| aacctgaggtccacagtatg \| \| tgctttccactcgtgctcct \| \| tttcatgtcctgggactcct \| \| tgggtgtaccccaaggtgat \| \| aaggcctcgaagcgacagat \| \| gaaggttctcattgttgtcg \| \| cacctcacacggttctcaat \| \| aagctgattggcgatgtgag \| \| acttgatcttttgcccttct \| \| agaggaaaggatacagcccc \| \| tcaggaaaagggactgagta \| \| ttgccttggctcacagcatc \| \| ccacccctgttgtgctttta \| \| aatgatgagtgacagacagg \| |
| *Oct4* intron (40 probes) | \| aaccttaaggccaagttcct \| \| --- \| \| aacaagagctcctatcagca \| \| tcgtgtaaaggtgactcatg \| \| aagggtgtccctttcttgtt \| \| tgtaggccatcagacactaa \| \| acaacaatcgctaagctgtc \| \| gggccatttaagatgtgaga \| \| tctccaactgctcctcaaaa \| \| agaaatggaggcagtcatct \| \| tctaagttgcagcgtgtgaa \| \| tatgagcaatagaacggcag \| \| actagagtgcgacagagaaa \| \| atccctctgttcagctctaa \| \| gtcccaaagtatgacacagt \| \| aagcaccattttttaccccc \| \| aaacttgactgaaggtgagc \| \| aatcgatcagatctgcacct \| \| tggagataaaactcccctac \| \| ttgcttacacttgctccaga \| \| acacagaaactggcacttag \| \| gcagtgtctttggcttttct \| \| aaaggattctctcggcttca \| \| ggcttttctgtctctaacag \| \| taacagatggccagttgagt \| \| gcatgcacacaccacaaaaa \| \| aagtagccaaatgtccatgc \| \| aagggctggggtaataagat \| \| cccaacctcttcagtaacaa \| \| tctgaggctaaagtagacag \| \| caaggaaaggtagaaaggct \| \| gtgcactcacagaatgatct \| \| actcgcaccttgttcttaag \| \| attaatgccttcctagggga \| \| ccaaaacttgtaatcgccct \| \| cacacctcaatgccatttca \| \| caaaatggctgtcggtttct \| \| tagtacacagtgatggttgg \| \| aaatcatctgactcaccctg \| \| gaaaacctacacagcacact \| \| aaacagggactcactaggaa \| |
| *Nanog* exons  (40 probes) | \| ggatgaaaaactgcaggcat \| \| --- \| \| tgaagaggcaggtcttcaga \| \| tctgaaacctgtccttgagt \| \| cctttggttttgaaaccagg \| \| acatggaaaggcttccagat \| \| ttgctgcaactgtacgtaag \| \| tgctaaaatgcgcatggctt \| \| tttaagcccagatgttgcgt \| \| tacgtaacaagatctgacgc \| \| ccaaagcctagagttaacac \| \| aaaaagactagcatgggtgg \| \| gagtagccaccatatcgtta \| \| gcaccttaataggtgaaagc \| \| ttaaactagtccagctggca \| \| cagacccttgtaagcaagaa \| \| tgggactggtagaagaatca \| \| atggaggagagttcttgcat \| \| aaccactggtttttctgcca \| \| ttgttccaagttgggttggt \| \| tccaaatcactggcagagaa \| \| gtcacagagtagttcaggaa \| \| ttggaagaaggaaggaacct \| \| aaccacatggtggctcacaa \| \| gagtatatgcacctcactgt \| \| acagtgtataccaagaccca \| \| atctgagctaccctcaaact \| \| acatagcagttactcttggg \| \| tctgtgcagagcatctcagt \| \| tcaggacttgagagcttttg \| \| ctgcttatagctcaggttca \| \| gaatcagaccattgctagtc \| \| aaagtcctccccgaagttat \| \| cctagtggcttccaaattca \| \| gtctcatatttcacctggtg \| \| gacagctacagtgtacttac \| \| aaggtcaggagttcaaatcc \| \| gcacttattcttgggaagga \| \| aaacctcacccctcaaaatg \| \| gttggccttgaacttattgc \| \| ggttcatcatggtacagtca \| |
| *Nanog* intron (48 probes) | \| ttcggggactgaattcctta \| \| --- \| \| gggtttccagaagagtgata \| \| ttatattgctccgtcctgtg \| \| aagctaggatgttaggtctc \| \| actgcttctgctggagaaaa \| \| ttgtttggggtttggaagga \| \| tttacaagcctgagtactgg \| \| acttacaaaggctatcccca \| \| ccctgaaagcagcttctaaa \| \| atttcctagatccagcagca \| \| cgtttctcttatccttgacc \| \| ttaaaatgatcccactgggg \| \| ccactgagtcagctatatct \| \| ggacttttatctcgcctaga \| \| ttctaagggatagggtctca \| \| ccgtctcaacaaatagagac \| \| cagcccgttttttctactct \| \| aacgtatcaccggtcaaact \| \| gaacatattccaaagagccc \| \| ccaaaaaaatggggtgctca \| \| ccaaaggttgagagaaatgc \| \| ctccagatgctagctataag \| \| aaaaaggggacacacacttc \| \| gctctacacacatgctctaa \| \| cctgcagtctagcaaataag \| \| ttcagcaagagacaagtgct \| \| gtcagagggtccagttaatt \| \| ccccacccccaatttttttt \| \| tgggacctttcatactctac \| \| atccaaagactcaggtttgg \| \| gcagaggatctagtctatgt \| \| ctgagatgggagaatttgag \| \| cacccgcttatgttaatgac \| \| ccggatctctatttcagact \| \| tactgaagacaccactcact \| \| gccatttgggcaaattgcaa \| \| gaactgctaagtgacatcca \| \| gacaatgagcttcagacctt \| \| cacttttcccacctccaaaa \| \| aattatgccatctgctggca \| \| acactgaagacatctgtgct \| \| ctagctcttcggttagcttt \| \| ttctgctagtacaagagcag \| \| cacagtcctgagtttagaca \| \| tgctgggtgaatagaatcct \| \| tacctctctaccttctgagt \| \| aagacagcacaagagcttag \| \| tagcacaaatctaaagcccc \| |
| *lacZ* (72 probes) | See (Skinner et al., 2013) |
